# Survival of HIV infected patients on maintenance hemodialysis in Cameroon: a comparative study

**DOI:** 10.1186/s12882-018-0964-8

**Published:** 2018-07-05

**Authors:** Marie Patrice Halle, Anais Mfoula Edjomo, Hermine Fouda, Hilaire Djantio, Noel Essomba, Gloria Enow Ashuntantang

**Affiliations:** 1 Faculty of Medicine and Pharmaceutical Sciences, Department of internal medicine-Douala general hospital Cameroon, University of Douala, Douala, PO Box: 4856, Cameroon; 2 Faculty of Medicine and Biomedical Sciences, Department of internal medicine- Douala general hospital Cameroon, Douala, University of Yaoundé I, Cameroon; 3 Higher Institute of Health Sciences, Université des Montagnes, Bangangte, Cameroon

**Keywords:** HIV patients, End stage kidney disease, Haemodialysis, Survival, Cameroon

## Abstract

**Background:**

There are conflicting reports on the impact of HIV in the era of combined antiretroviral (c-ART) on survival of patient with ESKD. We aimed to compare the one-year survival of HIV positive patients to that of their HIV negative counterparts with ESKD on maintenance haemodialysis in Cameroon.

**Methods:**

This was a retrospective cohort study conducted in the haemodialysis units of the Douala and Yaoundé General Hospitals. All HIV positive patients treated by maintenance haemodialysis between January 2007 and March 2015 were included. A comparative group of HIV negative patients with ESKD were matched for age, sex, co morbidities, year of dialysis initiation and haemodialysis unit. Relevant data at the time of haemodialysis initiation and during the first year of haemodialysis was noted. Survival was analysed using the Kaplan Meier and Cox regression hazard ratio estimator. A *p* value < 0.05 was considered statistically significant.

**Results:**

A total of 57 patients with HIV and 57 without HIV were included. Mean age was 46.25 ± 11.41 years, and 52.6% were females in both groups. HIV nephropathy (50.9%) was the main presumed aetiology of ESKD in the HIV group, while chronic glomerulonephritis (33.3%) and diabetes (21.1%) were the main aetiologies in the HIV negative group. At initiation of dialysis, the median CD4 count was 212 cell/mm^3^ (IQR; 138–455) and 77.2% were receiving c-ART. The proportion of patients who initiated dialysis with a temporary venous catheter was similar in both groups (*p* = 0.06). After one year on haemodialysis, survival rate was lower in the HIV positive group compared to the HIV negative group (61.4%/78.9%, HR: 2.05; 95% CI: 1.03–4.08; *p* = 0.042).Kaplan Meier survival curve was in direction of a lower survival in HIV positive group (*p* = 0.052).

**Conclusion:**

The one year survival of HIV positive patients on maintenance haemodialysis in Cameroon seems to be lower compared to their HIV negative counterparts.

## Background

Despite significant progress in the past two decades, human immune deficiency virus (HIV) infection remains a major cause of death and disease burden worldwide. In 2015, about 38.8 million people were HIV positive with 75% of them living in Sub-Saharan Africa (SSA) [1]. Chronic kidney disease (CKD) a common complication of HIV infection is associated with increased morbidity and mortality [2, 3]. Due to predisposing genetics polymorphism in the myosin heavy chain 9 and Apo lipoprotein L1 genes, black people have a 3 to 6 fold higher risk of HIV related renal diseases than white [4–8] and HIV- associated nephropathy (HIVAN) is the 3th leading cause of end stage kidney disease (ESKD) amongst black in the United States of America [9]. Impaired renal function has also been identified as a risk factor for death in the general and in HIV-infected populations [2, 10, 11]. The prognosis of HIV patients with ESKD was very poor and access to dialysis for these patients was limited in some countries before the introduction of combined antiretroviral treatment (c-ART) [12]. The poor outcome was mainly dependent on the severity of the HIV infection [10, 12–14].

C- ART is based on the association of three anti-viral drugs that inhibit the HIV replication. It was introduced in the care of HIV patients in 1997 and has improved the survival of these patients on renal replacement therapy (RRT) [15]. Reported results on survival of HIV positive patients compared to HIV negative one on haemodialysis are conflicting. In the USA survival of HIV patients with ESKD was lower compared to HIV negative patients [10, 16], while 2-year survival of treated HIV positive patients on haemodialysis was comparable to HIV-negative ones in France [17]. Despite the burden of HIV and the improve access to RRT and c-ART in most countries in sub Saharan Africa (SSA), data on survival of HIV patients on RRT are rare. A recent study in South Africa reported a similar survival between HIV positive and negative patients [18].

HIV infection is endemic in Cameroon with a population prevalence of 4.3% [19]. Access to c-ART is free and HIV does not constitute a contraindication to RRT. In Cameroon, about 8 to 10.8% of the haemodialysis population is HIV positive [20, 21]. Outcomes of patients on maintenance haemodialysis in Cameroon remain very poor [22, 23]. The impact of HIV positivity on patient outcomes on maintenance haemodialysis is unknown. This comparative study aimed to determine the survival of HIV infected patients with ESKD on maintenance haemodialysis in Cameroon.

## Methods

### Study setting

This was a retrospective cohort study carried out from January to Mars 2016 in the haemodialysis unit of the Douala and Yaoundé general hospitals, the two main tertiary hospital of the country. They are the referral hospitals for patients with kidney diseases of the littoral and centre region of the country. Both haemodialysis units are equipped with Fresenius® HD 4008S generators (Fresenius Medical Care, Hamburg, Germany), used synthetic polysulfone dialysis membrane and bicarbonate dialysate. No dialyzer reuse is practiced. The majority of patients underwent 2 dialysis sessions of 4 h per week. In Cameroon dialysis and c-ART is highly subsidized by the state and access to the treatment is not limited. HIV patients with ESKD are followed up by internist - nephrologist with a special training in management of HIV related diseases. The study received administrative authorization from the Douala and Yaoundé general hospital and ethical approval was obtained from the Douala University Ethics Committee.

### Patients

We included HIV infected patients who started maintenance haemodialysis between first January 2007 and 31th March 2015 in both hospitals. HIV patients with missing relevant data were excluded. A group of HIV-negative patients was then selected as matching controls and they were paired in a 1:1 ratio according to age, sex, date of dialysis initiation, and centre of dialysis. From medical records data collected initially were: socio demographic (age, sex, marital status), clinical (co morbidity, baseline nephropathy, vascular access and use of c-ART at dialysis initiation), para clinical (haemoglobin level, CD4 count, serum calcium and phosphorus level at dialysis initiation). While on dialysis we recorded opportunistic infections, outcome at one year (death, withdrawal or alive) and causes of death.

The clinical stage of HIV infection was based on the WHO classification [24]. ESKD was defined as initiation of chronic RRT for more than 3 months. The background nephropathy was mainly based on clinical arguments in the absence of renal histology data. Hypertension was defined as a blood pressure > 140/90mmhg or evidence from patient records that the patient was receiving antihypertensive treatment.

### Statistical analysis

Analysis was performed with Statistical Package for Social Science (SPSS) version 20.0. Categorical variables were reported as frequency and percentages and compared with Chi-square test or Fisher’s exact test when appropriated. Continuous variables were reported as mean ± standard deviation (SD) and median and inter quartile range (IQR) and their comparisons were done respectively by Student t-test and Mann Whitney U test. Survival analysis was done using the Kaplan Meier method (log rank test).ß probability of type II error was computed for borderline *p* value. Cox proportional hazard regression models were used to determine predictors of death. Basic models were adjusted for age, and then final models were further adjusted for age and all predictors with a *p* value < 0.1 in the basic models (diabetes, absence of c-ART at dialysis initiation). A *p* value < 0.05 was considered statistically significant.

## Results

During the study period, 67 HIV patients started dialysis in both centres, 10 were excluded for missing data. We included 57 HIV positive patients (21 in Yaoundé and 36 in Douala GH) and they were paired with 57 HIV negative patients according to sex, age, dialysis centre and date of dialysis initiation. Their socio demographic, clinical and biological profiles are summarized in Table 1. Mean age was 46.00 ± 11.4 years (*p* = 0.099, 52.6% were females (*p* = 1.00). HIV positive patients were more single (*p* = 0.004). Hypertension was the main co morbidity in both group and was more prevalent in HIV negative (89.5% versus 61.4%, *p* < 0.001), followed by diabetes (26.3% versus 15.8%, *p* = 0.168). The main presumed aetiology of ESKD was HIV (50.9%) in the HIV positive group, chronic glomerulonephritis (33.3%) and diabetes (21.1%) in HIV negative patients (*p* < 0.001).

**Table 1:** Baseline characteristic of the study population (*n* = 114)

Variables		HIV- *N* = 57	HIV + *N* = 57	p
Gender n (%)				
Female		30 (52.6)	30 (52.6)	
Male		27 (47.4)	27 (47.4)	1
Age (years)	Mean ± SD^a^	46.25 ± 11.41	46.00 ± 11.40	0.909
Matrimonial status n (%)				
Married		42 (73.7)	27 (47.4)	
Non married		15 (26.3)	30 (52.6)	**0.004**
Co morbidity n (%)				
History of hypertension		51 (89.5)	35 (61.4)	**< 0.001**
History of diabetes		15 (26.3)	9 (15.8)	0.168
Hepatitis B infection		3 (5.3)	4 (7.0)	1
Hepatitis C infection		8 (14.0)	8 (14.0)	1
Baseline nephropathy				
HIV^b^		0 (0.0)	29 (50.9)	
CGN^c^		19 (33.3)	8 (14.0)	
Unknown		11 (19.3)	11 (19.3)	
Hypertension		15 (26.3)	7 (12.3)	
Diabetes		12 (21.1)	2 (3.5)	**< 0.001**
Vascular access at initiation n (%)				
Temporary CVC^d^		45 (78.9)	52 (91.2)	
AVF^e^		12 (21.1)	5 (8.8)	0.066
Biologic Data	Mean ± SD^a^			
Haemoglobin		7.86 ± 1.98	7.12 ± 1.70	**0.045**
Calcemia		76.59 ± 14.78	73.64 ± 16.78	0.370
Phosphoremia		71.13 ± 28.84	67.88 ± 35.47	0.639
CD_4_ count *n* = 49				
Median (IQR^f^)		212 (138–455)	–	–
CD_4_ count<200cells/mm^3^ n (%)		23 (46.9)	–	–
Use of c-ART^g^ n (%)		44 (77.2)	–	–
Drug regimen n (%)(*n* = 44)			–	–
1st line		37 (84.1)	–	–
2nd line		6 (13.6)	–	–
Hospitalisation rate n (%)		10 (17.5)	12 (21.1)	0.635

^a^*SD* Standard deviation, ^b^*HIV* Human immune deficiency virus, ^c^*CGN* Chronic glomerulonephritis, ^d^*CVC* Central venous catheter, ^e^*AVF* Arterio- venous fistula, ^f^*IQR* Interquartile range, ^g^*c-ART* Combined antiretroviral treatment

Values in bold are significant (*p* < 0.05)

**Fig. 1:**
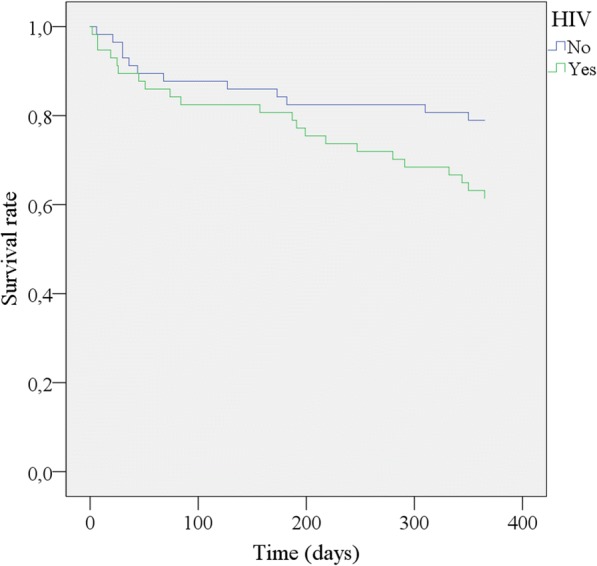
Kaplan Meier Survival curve; *p* value = 0.052 for log rank test, ß probability = 0.84

At dialysis initiation a temporary catheter was the main vascular access in both group with no difference (*p* = 0.066). Median CD4 count in the HIV group was 212cell/mm^3^ (IQR; 138–455) and 46.9% had a CD4 count < 200 cell/mm^3^, with only 77.2% of them on c-ART. Mean haemoglobin levels was significantly lower in the HIV-positive (*p* < 0.01). There was no difference between the group for serum calcium and phosphorus. Hospitalization rate was similar in the two groups (10 in HIV positive versus 12 in negative one, *p* = 0.63).After one year on dialysis global survival was 70.2% (80/114). The survival rate of HIV positive patients was lower (61.4%; 35/57) compared to negative one (78.9%; 45/57) with a twofold probability of death (HR: 2.05; 95% CI: 1.03–4.08; *p* = 0.042), (Table 2). The Kaplan Meier survival curve was in direction of a lower survival in the HIV positive group (Fig. 1)with a borderline *p* value (0.052) for long rank test and a high ß probability of type II error (0.84).The main causes of death were sepsis in both group, followed by tuberculosis and loss of follow up in the HIV group and haemorrhagic shock from gastrointestinal bleeding in HIV negative patients (Table 3).The main predictor of death in the HIV positive patients was the absence of c-ART at dialysis initiation. (Table 4).

**Table 2:** Mortality and survival rate

	All patients *N* = 114 n (%)	HIV- *N* = 57 n (%)	HIV+ *N* = 57 n (%)	HR^a^ (95% CI^b^)	*p* value
Mortality rate	34 (29.8)	12 (21.1)	22 (38.6)	2.05 (1.03–4.08)	**0.042**

^**a**^ *HR* Hazard ratio(Unadjusted univariate Cox regression analysis), ^b^: Confidence interval

Value in bold is significant (*p* < 0.05)

**Table 3:** Presumed causes of death

Causes of death	All patients *N* = 34 n (%)	HIV- *N* = 12 n (%)	HIV+ *N* = 22 n (%)	*p* value
Sepsis	14 (41.2)	6 (50.0)	8 (36.4)	0.440
Tuberculosis	4 (11.8)	0 (0.0)	4 (18.2)	0.273
Withdrawal from Dialysis	4 (11.8)	0 (0.0)	4 (18.2)	0.273
Gastro intestinal bleeding	4 (11.8)	3 (25.0)	1 (4.5)	0.115
Hemorrragic stroke	1 (2.9)	0 (0.0)	1 (4.5)	1
Pulmonary edema	1(2.9)	0(0.0)	1(4.5)	1
Cancer of the kidney	1(2.9)	0(0.0)	1(4.5)	1
Undetermined	5 (14.7)	3 (25.0)	2(9.0)	0.677

**Table 4:** Predictors of death amongst HIV positive patients (Cox regression analysis)

Variable	Basic models		Final models	
	HR^a^ (95% CI^b^)	p	HR^a^ (95% CI)	p
Age	1.02 (0.98–1.06)	0.284	1.02 (0.98–1.06)	0.452
Gender (female vs male)	1.00 (0.40–2.52)	0.993	1.09 (0.44–2.72)	0.857
Unemployed	1.24 (0.53–2.89)	0.619	1.95 (0.76–4.97)	0.165
Absence of nephrology follow up before 1st dialysis	1.14 (0.49–2.65)	0.757	0.93 (0.39–2.19)	0.863
Dialysis initiation with a temporary catheter	2.30 (0.31–17.09)	0.417	3.31 (0.42–25.94)	0.254
Hypertension	1.03 (0.43–2.48)	0.949	0.89 (0.33–2.42)	0.825
Diabetes	2.32 (0.87–6.19)	0.094	1.76 (0.64–4.85)	0.271
Hepatitis B	0.52 (0.07–3.90)	0.528	0.21 (0.03–1.81)	0.156
Hepatitis C	1.39 (0.44–4.39)	0.572	1.40 (0.43–4.61)	0.579
No cART^c^ at dialysis initiation	2.99 (1.27–7.06)	**0.012**	2.66 (1.09–6.50)	**0.031**

^**a**^ *HR* Hazard ratio, ^b^: Confidence interval, ^c^: *cART* Combined anti-retroviral treatment

Basic models are adjusted for age, and final models are adjusted for age and all predictors with a *p* value < 0.1 in the basic models (diabetes, absence of c-ART at dialysis initiation)

Values in bold are significant (*p* < 0.05)

## Discussion

This study aimed to evaluate for the first time the outcome of HIV infected patients on maintenance haemodialysis compared to HIV negative ones in a setting where c-ART is free and access to dialysis not limited. Our results showed that HIV positive patients on haemodialysis were relatively young adults, severely immune depressed. After one year on dialysis survival rate of HIV positive patients was lower compared to negative one, with a twofold higher risk of death. Survival curve was in direction of a lower survival in HIV group with a borderline *p* value and a high ß probability of type II error.

The number of people living with HIV/AIDS has steadily increase, with greatest prevalence and mortality rate in SSA [1]. In Cameroon HIV and ESKD are public health problem, and ESKD due to HIV remain a serious concern [18–21]. Combined ART is freely available since 2007. Haemodialysis, the only RRT available is subsidized to 95% by the State and access is not limited for HIV patients as it is the case in some SSA countries [25, 26]. The present study revealed that HIV patients on haemodialysis were relatively young mostly female and were severely immune compromised at the start of haemodialysis. This in accordance with others reported findings in the literature [27].

Haemodialysis and HIV infection are two risks factors that exposed HIV patients on maintenance haemodialysis to death [10]. The widespread of c-ART has improved the outcome of HIV positive patients worldwide [15, 19, 28, 29]. Survival of these patients with ESKD receiving RRT, has also progressively improved [15, 30]. Data on survival of HIV positive patients on RRT in SSA are scanty and inexistent in Cameroon. We found that one year survival rate of HIV positive patient was 61.4%. Our survival rate of HIV patients is lower than reported findings in the international literature that range from 74 to 95.2% in HIV positive patient receiving haemodialysis [10, 17, 18, 31–33]. In the USA one year survival of HIV-positive patients receiving chronic haemodialysis was estimated at 74% [10, 31]. In the study of Tourret et al. and Trullas et al. in Europe survival rate was 93.8 and 95.2% respectively [17, 32]. Tayebey et al. in Iran, found a rate of 75% [33], and Fabian et al. in South Africa recently reported a 100% survival rate of HIV-positive patients [18]. In the contrary our survival rate is higher compared to the studies of Zako et al. in South Africa (51%) [34] and Rodriguez et al. (46%) [31].

Reported results on survival of HIV positive patients compared to HIV negative one on haemodialysis are conflicting [6, 10, 17, 18]. In the present study, at one year survival rate of HIV infected patient was lower compared to HIV negative (*p* value 0.042) with a twofold risk of death in HIV positive patients. The Kaplan Meier curve was in direction of a worse survival curve in the HIV positive group, with a borderline p value (0.052) and a high beta probability of type II error probably due to the small sample size of our study population (57 patients) and the short duration of follow up (one year). This lower survival of HIV patients compared to negative one was also reported by Ramon et al. in Spain and Martinez et al. in Italy and in the USA. [10, 16, 35, 36] In the contrary our result is different from the reports of Tourret et al. in France [17] and Fabian et al. in South Africa [18] who found a similar survival between HIV-positive and HIV-negative patient.

The main predictor of death amongst our HIV patients was the absence of c-ART at dialysis initiation. Survival of HIV positive patients on haemodialysis has been shown to be correlated with earlier stage of the infection, younger age, higher CD4 counts, and treatment with c-ART [10, 12, 16, 37, 38]. Despite that our patients were young, and compared to the international literature our lower survival rate can be explain by the fact that patients were at late stage of the disease with severe immune depression (46.9% had CD4 count < 200 cell/mm^3^) and 1/4 of them were not on c-ART at dialysis initiation due to late referral of patients, a common situation in our setting [39]. These patients mostly arrived for the first time in life threatening condition imposing a start of emergency dialysis without c-ART. These drugs are not directly available in the centre, but have to be order after the patient’s file has gone through a therapeutic comity. Also dialysis was initiated on a temporary catheter that was reported to increased morbidity and mortality of patients on RRT [40, 41]. Hospitalization rate in this study was similar in both groups. This is contrary to the reports of Fabian et al. where HIV infected patients had a higher rate of hospital admission mainly due to vascular access infection [18].

We acknowledge some limitations in this study such as the small sample size and the short length of follow up (one year) that could influence survival. Also viral load of HIV positive patients was not available, nor CD4 count during follow up. Those test were not done routinely by patients due to the high cost as payment is out of pocket. Therefore we could not determine whether the HIV infection was controlled or not. Despite these limitations, this first study could serve as a baseline for future studies on this group of patients.

## Conclusion

We can conclude that in the c-ART area, survival of HIV-positive patients in Cameroon after one year on haemodialysis seems to be lower compared to their HIV-negative counterparts. This first result can serve as a basis for further studies with a large sample size and longer follow up period.

## Abbreviations

c-ART: combined Antiretroviral Treatment
CKD: Chronic Kidney Disease
ESKD: End stage Kidney Disease
HIV: Human Immune depressive Virus
HIVAN: HIV Associated Nephropathy.
RRT: Renal Replacement Therapy
SSA: sub Saharan Africa
